# Aquaporin-4-IgG Positive Neuromyelitis Optica Spectrum Disorder from Ethiopia: A Case Report

**DOI:** 10.4314/ejhs.v30i5.25

**Published:** 2020-09

**Authors:** Dereje Melka

**Affiliations:** 1 Department of neurology, college of health sciences, Addis Ababa University, Addis Ababa Ethiopia

**Keywords:** Neuromyelitis Optica, Aquaporin-4 antibodies, case report, Ethiopia

## Abstract

**Background:**

Neuromyelitis Optica spectrum disorder is an inflammatory disorder affecting the central nervous system), most commonly attacking the spinal cord or optic nerves. Limited cases of neuromyelitis optica have been reported in east Africa. Based on my review, if published, this would be the second published case of Neuromyelitis Optica spectrum disorder and the first published case of seropositive Neuromyelitis Optica spectrum disorder reported from Ethiopia. It signifies the need to have a high index of suspicion to promptly identify and properly treat these patients.

**Case Presentation:**

I am reporting a 32 years old female patient from Addis Ababa, Ethiopia, who presented with recurrent lower limb weakness and impairment of right eye vision of two-year duration. She was diagnosed based on Neuromyelitis Optica spectrum disorder diagnostic criteria, by having transverse myelitis, optic neuritis, confirmed by MRI imaging and high level of aquaporin-4-antibodies. Symptoms improved after providing five days of Methylprednisolone followed by low doses of corticosteroids and Azathioprine. The patient is now fully functional except for the right eye vision impairment.

**Conclusion:**

The patient described here signifies a classic manifestation of Neuromylitis Optica disorder with aquaporin-4-IgG occurring in Ethiopian woman. This case highlights the existence of Devic's disease within our setting and the need to properly diagnose this condition even in a resource-limited setting to avert disability.

## Introduction

Neuromylitis Optica (NMO) spectrum disorder is an inflammatory disorder affecting the central nervous system (CNS), commonly attacking the spinal cord or optic nerves (1). It is formerly assumed to be closely related to multiple sclerosis but more recently proven to represent a different clinical and pathophysiologic entity (1). This condition is also called Devic's disease or Devic's syndrome. NMO spectrum disorders include either recurrent longitudinally extensive myelitis (more than a three-vertebral segment spinal cord lesion seen in MRI) or recurrent optic neuritis (2).

The detection of antibodies to aquaporin-4, (2) a water channel found on astrocytic foot processes profoundly expressed in the optic nerves, brainstem, and spinal cord (1). It is a highly specific biomarker for NMO as well as for NMO spectrum disorders and is considered as an additional criterion supporting the diagnosis (2).

The International Panel for Neuromyelitis Optica Diagnosis (IPND) updated consensus diagnostic criteria incorporating relevant advances in 2015 (2). I am reporting this case considering that she fulfills this diagnostic criterion.

As NMO spectrum disorders carry substantial morbidity and, at times, mortality, early and accurate diagnosises followed by immediate initiation of therapy for both treatments of acute exacerbations and prevention of additional relapses are critical (1).

NMO is up to nine times more prevalent in women with a median age of onset of 39 years. In contrast to Multiple Sclerosis (MS), NMO is relatively common in non-whites (3). Limited cases of neuromyelitis optica have been reported in East Africa (4,5). In Ethiopia two cases have been reported, but only one of which was previously published (5).

The low expectation of such diseases in our setting, together with diagnostic difficulties, often leads to misdiagnosis and poorer outcomes in patients that could have benefited from available treatment. I believe that the awareness offered here may be valuable to several other resource-limited settings.

## Case Presentation

**History**: The patient was a 32 years old female from Addis Ababa, Ethiopia. She presented to one of the private clinics at Addis Ababa on January 18, 2017, with weakness on the right lower limb and numbness and tingling on the left lower limb of three days' duration. She had also associated urinary urge incontinence with a band like feeling on the right trunk below the breast fold. She had lost her right eye vision progressively seven months before the first visit. On the first visit, she was treated with five days of methylprednisolone intravenously and followed by oral prednisolone for 2 months and showed significant improvement. However, after six months of the first visit, the patient presented with another attack with weakness on the right lower limb of five days' duration with urinary urge incontinence. She was married and a mother of three children. Regarding past medical history, she was hypertensive on treatment with good control in the last 2 years. Otherwise, there was no personal or family history of diabetes or cardiac illness. No history of cough, night sweat, weight loss, or skin rash was recorded. She had no history of exposure to toxins and travel history out of Ethiopia..

**Physical examination**: On physical examination, she was noted to have a visual loss of the right eye with pale optic disc but no background change noted on fundoscopic examination. The right eye visual acuity was only light perception; left eye visual acuity was 6/6. Power on lower limbs was 3/5(right) and 4/5(left), reflex ¾ both on ankle and knee, plantar responses were up going bilaterally. Vibration and position sense decreased on the right body below the thoracic sixth vertebral spine and decreased pain, touch, and temperature sense on the left body below the thoracic sixth vertebral spine. The rest of the systemic and neurologic examination was unremarkable.

**Figure 1:**
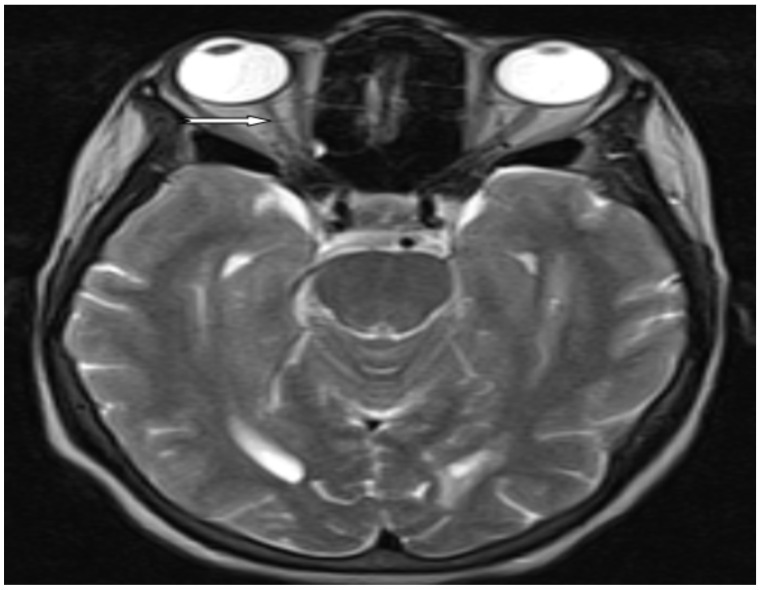
Magnetic resonance imaging studies. Axial T2-weighted Magnetic resonance imaging of the brain showed normal finding except for the presence of right side atrophied optic nerve

**Figure 2:**
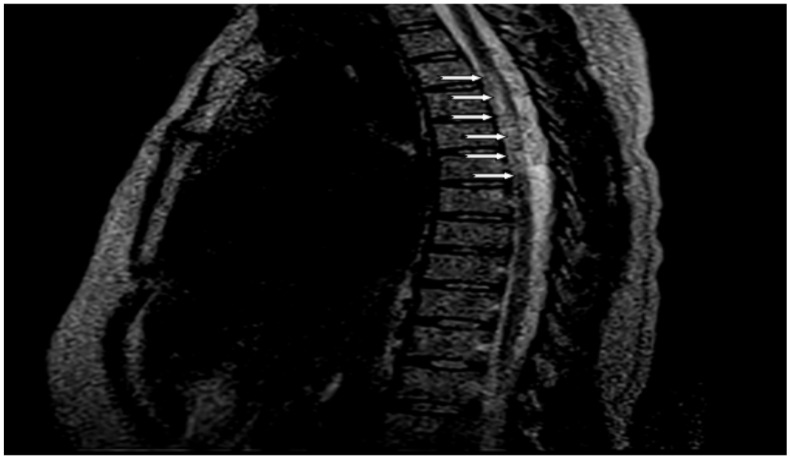
Sagittal T2-weighted Magnetic Resonance Imaging demonstrates ill-defined T2 bright signal intensities (myelitis) from T4 to T7 (white arrow)

**Figure 3:**
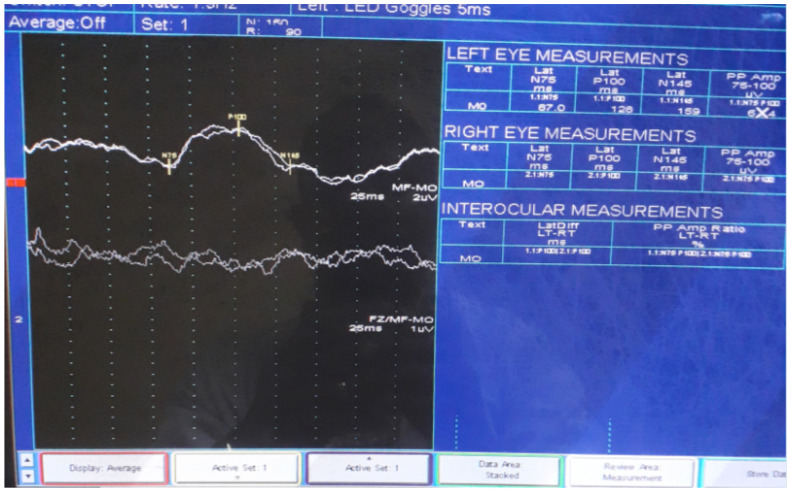
Visual evoked potential demonstrates moderately prolonged P100 latency on the left eye and absent response on the right eye

**Auxiliary examinations**: Her complete blood count, erythrocyte sedimentation rate, liver function test, renal function test, serum electrolytes, and plasma glucose levels were normal. Serum VDRL was non-reactive and serology for retroviral infections was also negative. Serum Antinuclear antibody (ANA) was also negative. Serum AQP4-IgG titer (using AQP4-IgG serologic assay techniques) was also determined on the second attack and reviled 1:200+++ (normal value <1:10). Chest X-ray and abdominopelvic ultrasound results were also normal. Her brain MRI finding was normal except for the presence of the right side atrophied optic nerve (Figure 1). Thoracic MRI (Figure 2) revealed hyperintense ill-defined lesions from the thoracic four (T4) to the thoracic seven (T7) spinal cords. However, the limitation of this case report was, contrast (gadolinium) was not administered during MRI study. Visual Evoked Potential (VEP) of the patient also revealed left side moderate demyelinating optic nerve neurophysiologic impairment (Optic neuritis, P100 latency of 128 milliseconds) and absent response on the right eye suggesting severe optic nerve neurophysiologic impairment (Figure 3). However, a cerebrospinal fluid analysis was not done considering that the case was a patient with classic NMOSD.

## Discussion

In contrast to Multiple Sclerosis (MS), NMO is relatively common in non-whites (3). According to my literature review, limited cases of published neuromyelitis optica have been reported in east Africa. This case shows the existence of NMO spectrum disorder with aquaporin-4-IgG occurring in Ethiopian patients.

My patient meets the International Panel for Neuromyelitis Optica Diagnosis (IPND) consensus diagnostic criteria 2015 (Table 1) (2). These criteria allow NMOSD diagnosis with the presence of at least 1 of 6 core clinical characteristics and detection of AQP4-IgG (2). Diagnostic necessities are strict for patients in whom AQP4-IgG is not detected or for whom testing is unavailable. Such individuals must experience 2 or more different core clinical characteristics (i.e., dissemination in space, affecting different neuroanatomic regions) and other supportive MRI features meant to enrich diagnostic specificity must also be present. The two required core characteristics may occur with a single clinical attack (e.g., classic Devic syndrome with simultaneous optic neuritis and acute myelitis with LETM) or multiple attacks (2). Meeting this criterion in my setting may still be challenging, particularly since serologic assays are not readily available, even if its absence does not prohibit the diagnosis and providing correct treatment. The previously reported case in Ethiopia was also based on clinical criteria where serum level of AQP4-IgG level was not determined. This makes my case the first NMOSD patient with serologic evidence of AQP4-IgG if published.

**Table 1:** International Panel for Neuromyelitis Optica Diagnosis (IPND) updated consensus NMOSD diagnostic criteria (2015) for adult patients

IPND updated consensus NMOSD diagnostic criteria(2015) for adult patients
**Diagnostic criteria for NMOSD with AQP4-IgG** 1. At least 1 core clinical characteristic 2. Positive test for AQP4-IgG using best available detection method (cell-based assay strongly recommended) 3. Exclusion of alternative diagnoses
**Diagnostic criteria for NMOSD without AQP4-IgG or NMOSD with unknown AQP4-IgG status** 1. At least 2 core clinical characteristics occurring as a result of one or more clinical attacks and meeting all of the following requirements: a. At least 1 core clinical characteristic must be optic neuritis, acute myelitis with LETM, or area postrema syndrome. b. Dissemination in space (2 or more different core clinical characteristics). c. Fulfillment of additional MRI requirements, as applicable. 2. Negative tests for AQP4-IgG using best available detection method, or testing unavailable. 3. Exclusion of alternative diagnoses.
**Core clinical characteristics** 1. Optic neuritis (Figure 3). 2. Acute myelitis (Figure 2). 3. Area postrema syndrome: episode of otherwise unexplained hiccups or nausea and vomiting. 4. Acute brainstem syndrome. 5. Symptomatic narcolepsy or acute diencephalic clinical syndrome with NMOSD-typical diencephalic MRI lesions. 6. Symptomatic cerebral syndrome with NMOSD-typical brain lesions.
**Additional MRI requirements for NMOSD without AQP4-IgG and NMOSD with unknown** **AQP4-IgG status** 1. Acute optic neuritis: requires brain MRI showing (a) normal findings or only nonspecific white matter lesions(Image 1), OR (b) optic nerve MRI with T2-hyperintense lesion or T1-weighted gadolinium enhancing lesion extending over >1/2 optic nerve length or involving optic chiasm. 2. Acute myelitis: requires associated intramedullary MRI lesion extending over ≥3 contiguous segments (LETM) OR >3 contiguous segments of focal spinal cord atrophy in patients with history compatible with acute myelitis (Figure 2). 3. Area postrema syndrome: requires associated dorsal medulla/area postrema lesions. 4. Acute brainstem syndrome: requires associated periependymal brainstem lesions.

Abbreviations: AQP4 = Aquaporin-4; IgG = Immunoglobulin G; LETM = Longitudinally Extensive Transverse Myelitis Lesions; NMOSD = Neuromyelitis Optica Spectrum Disorders

Patients with attacks of NMOSD develop significant disability if untreated (1). This is in contrast with individuals with multiple sclerosis who usually have relatively better recovery from attacks but develop a disability in the later, progressive stages of the disease (1). This is evidenced in this patient that she lost her right eye vision since she did not get timely intervention, but she had significant improvement and no disability on the subsequent extensive attacks (myelitis) because of early interventions (administration of immunotherapy). Therefore, a high index of suspicion is essential to make this diagnosis early and demonstrate the need for timely and prompt diagnosis to delay unfavorable consequences. This case is a leaving experience that emphasizes the importance of early intervention.

The patient described here signifies a classic manifestation of NMO spectrum disorder with aquaporin- 4-IgG occurring in this Ethiopian woman. The low expectation of such diseases in my setting, together with diagnostic difficulties, often leads to misdiagnosis and poorer outcomes. This case highlights the existence of Devic's disease within our setting and the need to properly diagnose this condition even in a resource-limited setting to avert disability by intervening timely. I believe that the awareness offered here may be valuable to several other resource-limited situations.
